# Use of an Environmental Pollutant From Hexavalent Chromium Removal as a Green Catalyst in The Fenton Process

**DOI:** 10.1038/s41598-019-49196-9

**Published:** 2019-09-06

**Authors:** Pricila Maria Batista Chagas, Aline Aparecida Caetano, Aline Auxiliadora Tireli, Pedro Henrique Souza Cesar, Angelita Duarte Corrêa, Iara do Rosário Guimarães

**Affiliations:** 1 Laboratório de Catálise Ambiental e Novos Materiais, Departamento de Química, Universidade Federal de Lavras, CEP 37200-000 Lavras, MG Brazil; 2 Laboratório de Química, Departamento de Química, Universidade Federal de Itajubá, Centro Universitário de Itajubá, CEP 37500-903 Itajubá, MG Brazil; 3 Laboratório de Bioquímica, Departamento de Química, Universidade Federal de Lavras, CEP 37200-000, Lavras, MG Brazil

**Keywords:** Pollution remediation, Environmental impact

## Abstract

The present study refers to the use of an environmental pollutant generated during the removal of hexavalent chromium from aqueous media. This pollutant is a material with catalytic properties suitable for application in the oxidative degradation of problematic organic compounds. The material, initially used as an adsorbent, is a composite prepared by modifying the crystalline phases of iron oxides together with the chitosan (CT-FeCr). Chemical and morphological characterizations of the materials were performed using SEM analysis coupled with EDS, XRD and DSC. The CT-FeCr beads were used in the degradation of methylene blue dye (MB) and showed excellent degradation potential (93.6%). The presence of Cr on the surface of the catalyst was responsible for the increase in catalytic activity compared to the CT-Fe and pure magnetite materials. The product of the effluent treatment and the presence of the catalyst itself in the environment do not pose toxic effects. In addition, the CT-FeCr beads showed catalytic stability for several consecutive reaction cycles with possible technical and economic viability. The concept of “industrial symbiosis” may be applied to this technology, with that term relating to the reuse of a byproduct generated in one particular industrial sector by another as a raw material.

## Introduction

Industrial wastes, byproducts or residues generate environmental benefits and economic value and reduce the burden on society if they can be reused, remanufactured or recycled as the feedstock to another process. The reduction of waste along all stages of a product life cycle, including the after-use stage of the product, contributes to environmental sustainability, as well as economic and social sustainability^1^. The “industrial symbiosis” concept offers an innovative approach involving interindustrial recycling, whereby one industry takes the waste from another as a raw material. This approach reduces the value of inputs into the system as a whole, as well as the quantities of resources extracted from the environment^1,2^.

The incorporation of this model in processing industries is still a limited reality. For instance, the waste from the tanning industry is an important source of heavy metal pollutants, especially chromium. Only 60–70% of the applied chromium reacts with the processed materials; therefore, approximately 30–40% is released as solid and liquid wastes^3,4^. Cr^3+^ and Cr^6+^ species are the most common and stable chromium species found in effluents. The two forms have different chemical, epidemiological and toxicological properties^5^. Currently, these wastes have no commercial value and are stored in facilities that require large financial investments and constitute a risk to the environment^4^. In the current context, residues rich in potentially toxic substances such as heavy metals can be used as raw materials in order to produce ceramic pigments and catalysts^6^.

Based on this scenario, the present study coherently and cyclically organizes the recovery of trace elements or heavy metals, initially considered contaminating agents in an aqueous medium. The material initially used for metal adsorption is a composite prepared by modifying the crystalline phases of iron oxides together with chitosan. The chitosan itself is a low-cost oxybiodegradable biopolymer and a waste from the fishing industry, which can function as a stabilizing and dispersing agent to improve the catalytic activity of iron oxides^7–9^.

The adsorption processes for the removal of heavy metals are already well-known in the literature^10,11^. However, there is little current concern regarding the contamination cycle, such as what to do with the generated environmental pollutant, i.e., the contaminated adsorbent remaining after saturation and loss of activity of its materials. A process is often created that merely discusses the contaminant phase transfer and does not promote environmental remediation as a working premise. The removal of hexavalent chromium by a strong and irreversible adsorption process on the CT-Fe material surface makes viable use of this material a second time as a catalyst in the Fenton degradation.

Recent studies show that the catalytic activity of iron oxides is strongly influenced by the presence of different metal atoms in their structure, which can promote greater generation of radicals and increase the efficiency of the oxidation of organic compounds^12–15^. The synergistic and/or cooperative effects of bimetallic catalysts are among the most fascinating aspects of catalysis research. These catalysts are generally composed of an active parent species and the so-called promoter^16,17^. For example, the removal of hexavalent chromium by a strong and irreversible adsorption process on the CT-Fe material surface allows this material to be used a second time as a catalyst in the Fenton degradation. In this context, the chromium that once acted as an antagonist to the environment now has a fundamental role as a promoter for the activation of iron in the formation of hydroxyl radicals and oxidation of organic contaminants. Chromium has interesting characteristics for application in catalysis, such as: (*i*) different oxidation states, which may coexist depending on the reaction conditions and (*ii*) high reactivity for the activation of H_2_O_2_ in advanced oxidation processes (AOPs)^18–21^. Among the AOPs, Fenton-type processes have been highlighted because it is possible to obtain hydroxyl radicals from oxidants and catalysts with strong environmental appeal, such as hydrogen peroxide, a purely green oxidant, associated with some form of iron immobilized and/or conjugated to a second metal element^15,22,23^.

In this study, an added step was proposed for the adsorption cycle previously performed to create a continuous, closed-loop adsorption-catalysis process in which the chromium removed by adsorption in a first stage becomes a promoter in the catalytic cycle in the next step. In this way, the symbiotic relationships described are intended to provide substantial environmental benefits. The CT-Fe composite is formed by direct incorporation of Fe^2+^ into the soluble chitosan gel. The polymer structure is regenerated for the production of nanostructured magnetic iron oxides CT-Fe in a spherical structure. The CT-Fe beads are used as adsorbents in the removal of aqueous Cr^6+^. The composite material composed of CT-Fe, now with chromium immobilized by irreversible adsorption in its structure (CT-FeCr) is used in Fenton processes for the degradation of organic compounds. We optimized parameters such as temperature and the amount of H_2_O_2_ and assessed a study on the reuse of beads and regeneration as materials with catalytic properties. Once the possibility exists that this catalyst may release chromium into the environment, there is considerable concern regarding its biosafety. Hemolysis is one of the minimum parameters to be assessed in order to assure the safety of substances that may be in contact with animal organisms. In this study, a hemolysis assay was performed to assess the safety of the catalyst supernatant.

## Methods

### Catalyst preparation

The chitosan solution was prepared by solubilizing 3.5 g of chitosan (CT) (low molecular weight, obtained from Sigma-Aldrich® with a degree of deacetylation 80.7% ± 1.35) in 100 mL of acetic acid solution (2% v/v) with stirring for 1 h^24^. Then, 1.40 g of Fe^2+^ (CT-Fe 40% w/w) was incorporated into this solution using the reagent FeCl_2_.4H_2_O. The mixture was stirred until completely solubilized.

For the production of beads, the above mixture was dripped into a 2 mol L^−1^ NaOH solution, immediately coagulating a gel in the form of spherical beads. The chitosan solution was also dripped in order to obtain the pure chitosan beads. The beads remained for 16 h in the 2 mol L^−1^ NaOH solution and then were washed with distilled water until the solution was neutralized. Subsequently, the beads were oven-dried at 60 °C.

After obtaining the CT-Fe beads, they were used in the removal of aqueous Cr^6+^. A total of 500 mg of CT-Fe beads were added to 500 mL of 100 mg L^−1^ Cr^6+^ solution with stirring for 24 h. After adsorption, the content of residual chromium in the solution was determined by the colorimetric method, using 1,5-diphenylcarbazide at 540 nm^25^. The dosage of total Cr was determined after Cr oxidation in acidic medium with potassium permanganate at high temperature. The dosage of Cr^3+^ was calculated by the difference between total Cr and Cr^6+^ in the solution. The beads then were washed and dried at 60 °C, resulting in the material used in this study and designated as CT-FeCr. The magnetite particles were synthesized according to the classical precipitation method proposed by Cornell and Schwertmann^26^, with modifications.

### Characterization of materials

The material morphology was performed using an EVO MA15 instrument with an Oxford detector operating with a 20-kV electron beam coupled to the energy dispersive spectroscopy (EDS). The nitrogen adsorption-desorption isotherms were obtained at 77 K in a Quantachrome instrument (NOVA1200). Samples were previously degassed for 12 h at 100 °C. The specific surface area was calculated using the BET equation. The pore-size distribution was calculated based on the N_2_ adsorption isotherm using the Horvath-Kawazoe (HK) method. The beads were characterized by X-ray diffraction (XRD) using a Rigaku Geigerflex X-ray diffractometer equipped with a graphite monochromator and CuKα radiation (1.5406 Å) at a current of 40 mA and voltage of 45 kV. Differential scanning calorimetry (DSC) measurements were performed at 25–390 °C using a DSC Q20 2151 (TA Instruments) at a heating rate of 10 °C min^−1^ under an air atmosphere.

### Catalytic reactions

Initial optimization tests of the experimental parameters for the oxidation of methylene blue were carried out in 30 mL flasks containing 50 mg L^−1^ solution of a model molecule (methylene blue (MB) dye), 30 mg of the materials (CT-Fe, CT-FeCr, and Fe_3_O_4_), 300 μL of 30% H_2_O_2_ (producing a concentration in the reaction medium of 97.8 mmol L^−1^) with stirring in a temperature-controlled bath (initial temperature 25 ± 0.5 °C) and samples were withdrawn throughout 240 min. The degradation efficiency of the MB dye was monitored by UV-visible spectrometry (λ = 665 nm), and the dye concentration was calculated using an analytical curve. Prior to degradation, the reaction mixture was stirred until adsorption equilibrium was reached (no significant adsorption within 24 h was detected by the materials). Controls were performed with the dye and H_2_O_2_ in the same concentrations as in the degradation tests but without the presence of the catalysts.

The reaction parameters, such as the effect of temperature on the kinetics (25, 40, 40 and 60 °C, held in a thermostatic bath) and the H_2_O_2_ concentration (13.0 to 97.8 mmol L^−1^) on the degradation of methylene blue were also optimized.

To evaluate the technical viability of the material, the catalyst reuse process was studied. This study was performed with 30 mL of the standard organic compound solution at a concentration of 50 mg L^−1^, 30 mg of the CT-FeCr beads, 75 μL of 30% H_2_O_2_ with constant magnetic stirring for 60 min at the preset temperature of 60 °C. The beads were then filtered, washed with distilled water and placed again under the same initial reaction conditions. This cycle was performed until the activity was finally reduced to approximately 30% of the initial activity obtained in the first reaction cycle.

At the end of each degradation cycle of the model compound, leaching tests were performed on the supernatant. Quantification tests were performed for total Cr using the colorimetric 1,5 diphenylcarbazide method^25^ and for iron using the colorimetric orthophenanthroline method^27^. The active phase of the leaching test was also performed on the supernatant after dye degradation^28^. Thereafter, the remaining solution was heated in order to eliminate any possible residual H_2_O_2_. A dye load (up to a concentration of 50 mg L^−1^) together with the H_2_O_2_ was added to the solution and placed under the same initial reaction conditions with stirring for 3 h at 60 °C.

The regeneration study of the CT-FeCr catalyst was performed at the end of six reaction cycles. For regeneration activity, 100 mg of the beads was immersed in 5 mL of ethyl alcohol (P.A.) for 4 h with magnetic stirring. The beads were filtered and dried at 60 °C for 12 h. After this period, the material was reused in a new catalytic cycle.

### Toxicity-hemolysis assay

To evaluate the toxicity of the supernatant after the degradation of methylene blue by the CT-FeCr beads (0.01 g L^−1^ CT-FeCr/solution of MB 50 mg L^−1^, temperature 60 °C, contact time 60 min, 13 mmol L^−1^ H_2_O_2_), a hemolysis assay was performed according to ASTM practice guide F 756 with modifications. Briefly, the blood of three healthy individuals was collected on three different occasions in tubes containing citrate buffer. The tubes were centrifuged at 700 g for 15 min to precipitate the erythrocytes. Part of the plasma was removed, leaving only a small quantity containing the volume of the red blood cells. Part of this erythrocyte precipitate was added at a concentration of 1% to a volume of PBS solution (1.15 mmol L^−1^ of NaH_2_PO_4_.H_2_O, 4 mmol L^−1^ of Na_2_HPO_4_.H_2_O and 0.15 mol L^−1^ NaCl, pH 7.35). A total of 1 mL of the prepared solution was added to 2 mL capped tubes. Afterwards, samples containing 50, 100 and 150 µL of the 5 mg L^−1^ chromium standard solution and the catalyst supernatant (under experimental conditions) was added to the PBS to reach a final volume of 300 µL. The samples were placed in contact with the erythrocyte solution for 4 h. The results were recorded at 540 nm. Controls containing only PBS (C-/mechanical hemolysis), and *Bothrops jararacussu* (C + 40 µg), were performed. Total hemolysis was prepared in ultrapure water to induce maximum hemolysis. The hemolysis percentage was assessed using the following equation:

$$Hemolysis( \% )=\frac{Sample\,Abs-Mechanical\,Hemolysis\,Abs}{Total\,Hemolysis\,Abs}\times 100$$

The assay was carried out in triplicate and repeated three times. The data was analyzed using ANOVA. The means test was performed using the Scott-Knot method.

The human blood used for the test of hemolysis was obtained from healthy volunteers who had not used any medication for a period of 30 days before collection. The blood was collected by venipuncture in tubes containing heparin for the hemolytic activity. The protocols for the trials with human cells or blood components were evaluated and approved by the Ethics Committee on Human Research (COEP) at the Federal University of Lavras under the registration number CAAE: 64329117.9.0000.5148. All the proceedings were performed in accordance with relevant guidelines and regulations of the COEP (Federal University of Lavras) ethics committee. All participants are over 18 years and in possession of its mental faculties. The use and publication consent were given by all participants (Informed Consent Form Statement).

## Results

All assays performed in this study used the CT-FeCr derived from a simulated effluent treatment process containing hexavalent chromium in aqueous solution. The CT-Fe beads used in the report^29^ were used until the surface was saturated with chromium. The saturation was standardized as a point where no further trace element removal was possible by the CT-Fe beads. After this process, the saturated CT-Fe beads, now named CT-FeCr, were evaluated as for their catalytic properties.

### Characterization of materials

**Figure 1:**
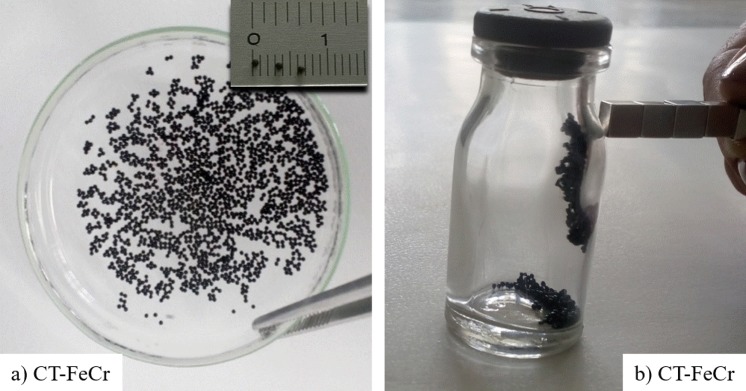
Photographic images of CT-FeCr beads (**a**) beads, and a dry bead of 1.0 mm diameter is shown in the insert, (**b**) magnetism.

Figure 1 shows the photographic images of the spherical CT-FeCr catalysts. The dry beads of CT-FeCr appear with dark and almost black coloration, typical of iron oxide in the magnetite phase, Fe_3_O_4_ (Fig. 1a). It can be observed in the upper right insert of Fig. 1a that this material presents a millimetric size distribution, approximately 1.0 mm diameter. This material is magnetic (Fig. 1b), which makes the recovery process easier and simpler in the reaction medium.

**Figure 2:**
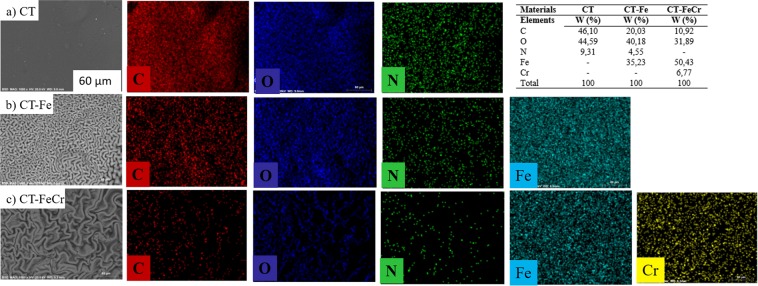
Scanning electron microscopy (SEM) and energy dispersive spectroscopy (EDS) of (**a**) Chitosan (CT) (60 μm); (**b**) CT-Fe (60 μm); (**c**) CT-Fe after removal of Cr^6+^ (60 μm).

Figure 2 shows the surface morphology of the beads as obtained by scanning electron microscopy (SEM) and the results of the analysis by chemical mapping of the main elements that constitute the beads, i.e., C, N, O, Fe and Cr, performed with energy dispersive spectroscopy (EDS) coupled to SEM. In the enlarged image of the surface of the pure CT bead (Fig. 2a), a smooth and homogeneous structure, with a regular morphology, was observed. In this polysaccharide, the SEM image showed some small cracks but there was no visible hard deformation of the surface. This homogeneous surface is probably related to the well-ordered arrangement of the chains of this polymer. The mapping of the element nitrogen on the CT bead surface, shown in green, reflects a uniform distribution across the material surface. In addition to that, the oxygen and carbon elements found in the chitosan structure also presented a uniform distribution, which supports the theory proposed from the chemical-structural analysis to affirm the morphological continuity of the chitosan beads.

**Figure 3:**
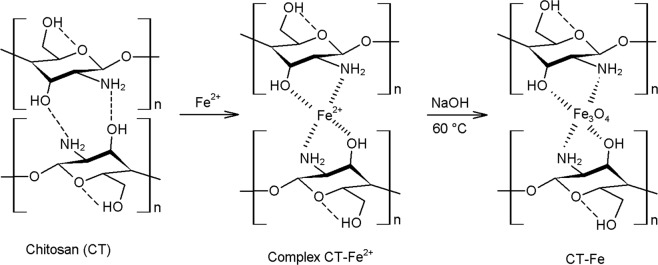
Scheme of synthesis of the CT-Fe beads. The magnetite is formed during the beginning of nucleation under alkaline conditions.

The presence of symmetrically distributed functional groups in the chitosan structure, such as -NH_2_ and -OH, provided a structural regularity of the polymer chain due to strong inter- and intramolecular hydrogen bonding interactions shown in the scheme available in Fig. 3.

Figure 2b depicts the metal-organic structures of the hybrid composite material, synthesized by the iron oxide dispersion in the chitosan network (CT-Fe). The SEM image clearly shows that the addition of iron has a strong effect on the final morphology of the hybrid composite. The formed iron oxide gives a highly roughened surface to the beads with a rather irregular exterior, showing more protuberant areas interspersed with small valleys, rich in grooves that give a rough appearance to the beads. The chemical mapping by EDS shows the homogeneous dispersion of the element Fe in blue throughout the surface of the hybrid materials, evidencing a well-dispersed formation of the oxide throughout the polymer structure. Based on the scheme of Fig. 3, it can be observed that the synthesis of the CT-Fe beads breaks the intermolecular hydrogen interactions. Thus, the metal, which acts as a Lewis acid, can occupy the bonding site of the bases, causing a morphological change in the material, evidenced by the removal of the typical hydrogen forces in the chitosan, as shown in Fig. 3. Thus, in the presence of Fe^2+^, the hydrogen bonds among the polymer chains are broken and the new metal bonds with the -NH_2_ and -OH groups lead to the formation of the chitosan/Fe complex. Once present in strongly alkaline medium, the iron is converted to the oxide form (Fig. 3).

Notably, the roughness present on the CT-Fe bead surface and the increased degree of disorder observed in the morphology positively contributed to the possible optimization of adsorption and the catalysis process. The hybrid material was applied with very satisfactory results in the removal of aqueous Cr^6+^. The CT-Fe beads showed a high capacity of removal of the metallic element (112.0 mg g^−1^) in an irreversible chemical process in which the chromium is firmly connected to the carbohydrate structure^29^.

The concept that the incorporation of the metallic element was responsible for disordering the chitosan structure by the removal of chains strongly bound by hydrogen bonds was strengthened when the surface profiles of beads after Cr^6+^ adsorption were observed in aqueous medium (Fig. 2c) compared with the CT-Fe surface (Fig. 2b). The cracks present in the outer portion of the material are more pronounced, and the valleys are more prominent. The Cr mapping (in yellow) shows that analogous to Fe, this metal element is also dispersed throughout the CT-FeCr bead surface.

**Figure 4:**
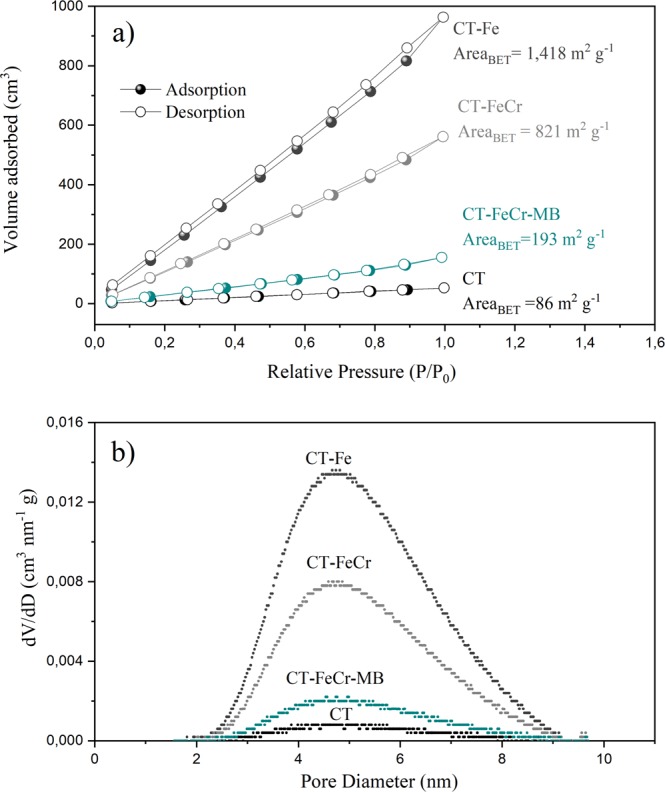
(**a**) Adsorption-desorption isotherms of N_2_ at 77 K and BET specific surface area; (**b**) pore-size distribution of the chitosan beads (CT); CT-Fe; CT-FeCr and CT-FeCr-MB.

To evaluate the effect of the roughness formed in the highly organized surface of the beads, the material was characterized by isothermal analysis of nitrogen adsorption/desorption. The extension of the specific surface area was calculated according to the BET equation. The adsorption/desorption curves of N_2_ and the pore-size distribution of the CT, CT-Fe, CT-FeCr and CT-FeCr-MB materials are shown in Fig. 4. The progressive increase of the adsorbed N_2_ volume over the entire P/P_0_ range can be attributed to the mesoporous nature of the material. The slight slope observed at a high relative pressure (p/p_0_ > 0.8–1) can be attributed to mesoporous capillary condensation^30^. The materials had pore diameters in the 2–9 nm range, with an average pore size of 5 nm (Fig. 4b), which characterizes them as mesoporous^31^.

The material isotherms resemble the type IVa isotherm model according to the standard classification of the International Union of Pure and Applied Chemistry (IUPAC). In this isotherm, the capillary condensation is accompanied by hysteresis, being that the hysteresis loops are not well defined and are closer to the H3 type in the present study. Loops of this type are given by nonrigid aggregates of plate-like particles^31^.

The values of the specific surface area (m^2^ g^−1^) of materials were calculated using the BET equation (Fig. 4a). It is important to emphasize that physisorption N_2_ isotherms were made in duplicate with two standard samples (Al_2_O_3_) between the analyses. The formation of iron oxide in the chitosan matrix caused a very relevant increase in surface area (1418.0 m^2^ g^−1^). This value is almost 17 times higher than that found for the chitosan sample (85.86 m^2^ g^−1^) and corroborates with the images obtained by SEM (Fig. 2a,b) in which a significant change in chitosan morphology was observed in the presence of metal. The highly roughened and irregular appearance of the CT-Fe hybrid composite has elevated the specific surface area and the adsorbed N_2_ volume of the materials. This increase favors adsorption processes, since the number of active sites available on the material surface also increases. In the same way, the adsorption of chromium ions by the CT-Fe composite reduced the surface area (820 m^2^ g^−1^), suggesting that the available active sites were occupied by the metal, reducing the contact surface. Moreover, the Cr can form cross-linked hydrogen bonds, which decreases the possibility of diffusion of the N_2_ probe molecule.

It is important to note that the surface specific area of the hybrid composites (CT-Fe and CT-FeCr) was extremely high in comparison to the bulk iron oxides such as magnetite (10–70 m^2^ g^−1^)^32–34^. This structural modification of the iron oxide increases both the adsorbent and the catalytic properties of the material. The CT-FeCr catalyst showed a decrease in its surface specific area after the degradation of the methylene blue dye (CT-FeCr-MB). This result is discussed in the catalyst reuse section (subitem 3.2.3).

**Figure 5:**
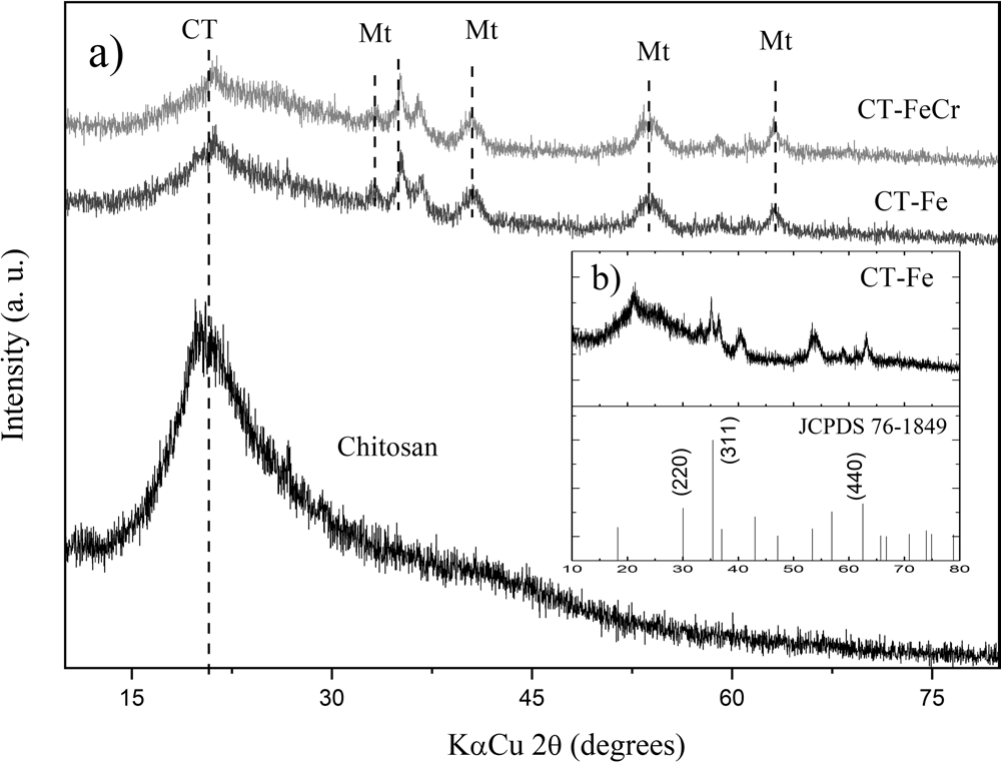
X-ray diffraction (XRD), chitosan beads (CT), CT-Fe beads (40% m/m); CT-FeCr and Fe_3_O_4_ reference data (JCPDS 76-1849).

Figure 5 shows the results of the microcrystalline characterization of the hybrid composites by XRD. The formation of the structured iron oxide in the chitosan matrix was identified as magnetite (Mt) with the analysis of the lattice parameters obtained by X-ray diffraction (Fig. 5a,b).

The pure CT diffractogram shows a reflection signal at 2θ = 20°, characteristic of semicrystalline polymers. The amplification of this signal can be attributed to a structure with a low degree of crystallinity and is directly related to the strong intra and intermolecular interactions, characterized by the hydrogen bonds formed among the chitosan functional groups^35^. In the chitosan XRD profile after the interaction with the metallic ions (Fe/Cr), a reduction in crystallinity was observed, thus representing a more amorphous structure. This behavior refers to the formation of CT-metal complexes and the new organization in the polymer structures after interaction with the metal ion, where the diffraction signals decrease in intensity (Fig. 5a). Since the metal broke the hydrogen bonds, which previously held together the chitosan chains, chelates were formed with -NH_2_ or -OH, causing distortions in the crystal structure (Fig. 3)^36^.

After the incorporation of Fe, there were new diffraction signals at approximately 2θ = 18.3, 35.4, 37.0, 43.0, 47.1, 53.4, 56.9, 62.5, and 65.7°, which, according to the indexed database, are characteristic of the Fe_3_O_4_ cubic structure (JCPDS 76-1849, Fig. 5b). The interaction between chitosan and Fe causes a displacement in signals when compared with pure Fe_3_O_4_ materials^37^. The magnetite phase found by the crystalline profile of the hybrid composite suggested that Fe^2+^ was partially oxidized to Fe^3+^ during the synthesis of the composite beads. The primary crystallites or Fe_3_O_4_ nanocrystals are formed in the polymer network of the chitosan at the beginning of nucleation under alkaline conditions (Fig. 3).

**Figure 6:**
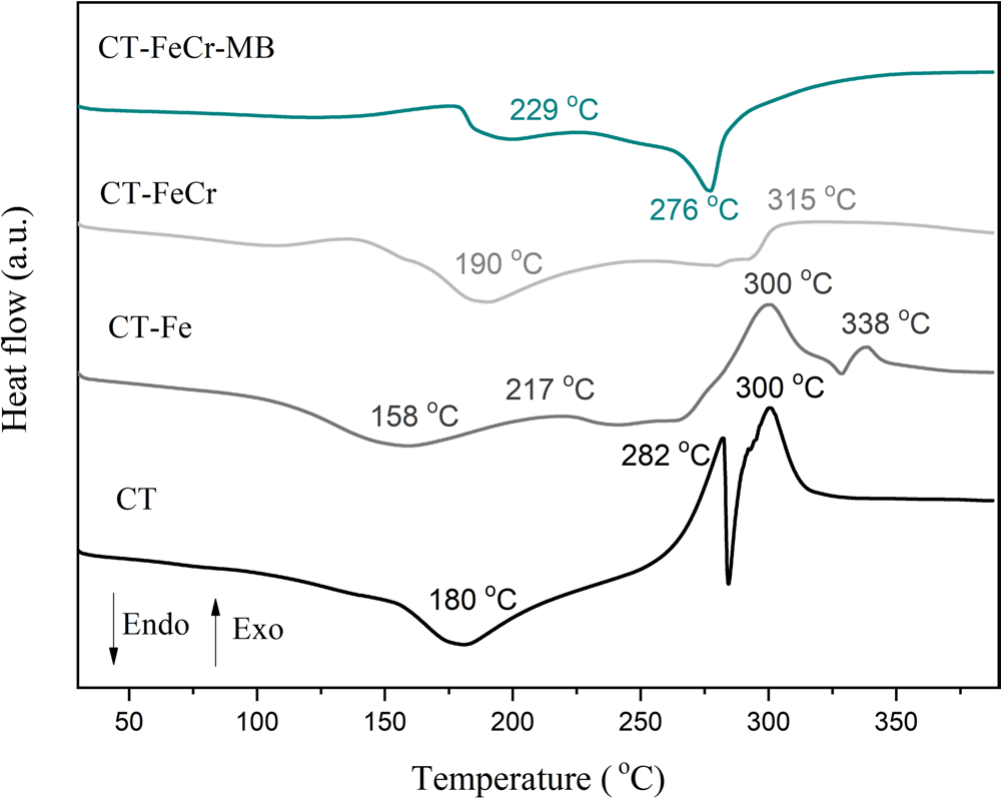
DSC of chitosan beads (CT), CT-Fe, CT-FeCr and after degradation of methylene blue CT-FeCr-MB (heating rate of 10 °C min^−1^ under a synthetic air atmosphere).

In Fig. 6, the DSC results for CT, CT-Fe, CT-FeCr and CT-FeCr-MB are presented. Some temperatures associated with the endothermic and exothermic events were identified in the DSC curves.

The results of the DSC analysis are the following. For CT, three thermal events were observed: The first was an endothermic signal before 200 °C, corresponding to the evaporation of residual water absorbed due to the hydrophilic nature of its functional groups. The events with exothermic signals at 282 °C and 300 °C correspond to the oxidative degradation of chitosan polymer chains. The first signal refers to the loss of -NH_2_ groups and the second to interchain reactions of polymer and acetyl groups.

In relation to CT-Fe, it was observed that the endothermic event occurred at a temperature lower than that of the chitosan, indicating a greater ease in dehydration, which may be associated with the amorphous nature of the polymer. Such an effect is probably due to the breakdown of hydrogen bonds in the presence of the metallic element. The formation of magnetite in the composite is evidenced by the exothermic process that took place at a temperature close to 217 °C, relative to the oxidation of Fe^2+^ to Fe^3+^ and corresponding to the conversion of magnetite to maghemite. The broad exothermic signals at 300 °C and 338 °C refer to the sum of different events happening in this temperature range. In this range occur both the degradation of chitosan polymer chains, as described for this carbohydrate alone, and the phase transition of maghemite oxide to its final phase, hematite. The presence of metals induced the reorganization of polymer chains in the beads and affected the thermal degradation process; thus, these events occur at higher temperatures. This effect can be attributed to the variation in the amine groups of chitosan, in which the connections for the formation of metal/chitosan complex occur^38^. Thus, in the curves of the CT-FeCr and CT-FeCr-MB materials, the oxidative degradation of polymer chains must be occurring at temperatures above 350 °C. In the curve referring to the CT-FeCr-MB material after dye degradation, the endothermic signal at 276 °C can be attributed to thermal desorption of intermediates that were produced and adsorbed on the catalyst surface during the catalytic reaction of the degradation. This result is particularly relevant because it can represent one of the ways to regenerate the catalyst surface with thermal treatment.

A plan following a “circular economy” model is part of a cyclical process that aims at the creation of large self-sustaining industrial clusters, with minimization of residues and maximum atomic efficiency. In such a plan, the composites that were considered as environmental liabilities resulting from hexavalent chromium remediation were used as materials with catalytic properties for oxidative degradation of organic compounds, based on the MB probe molecule. The results of the optimization of the oxidation process are shown in the following steps.

### Catalytic performance in the Fenton process – experimental design

#### Temperature effect

**Figure 7:**
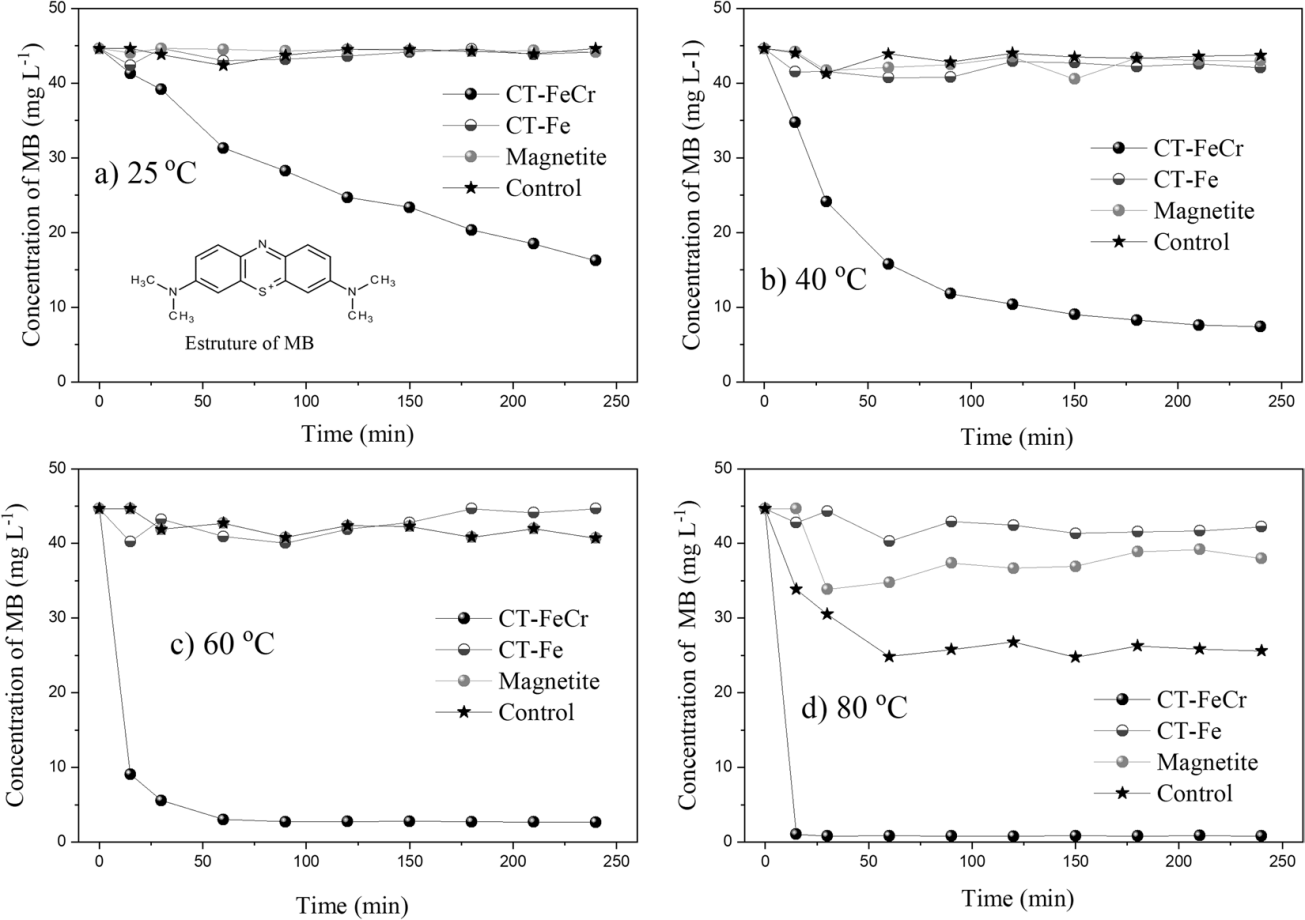
Effect of temperature on the oxidative degradation of methylene blue - MB (1 g L^−1^ CT-FeCr, CT-Fe, magnetite and control (H_2_O_2_ + dye and without catalyst)/MB solution 50 mg L^−1^, pH 6.0, H_2_O_2_ 97.8 mmol L^−1^) (**a**) 25 °C; (**b**) 40 °C; (**c**) 60 °C; d) 80 °C.

The temperature effect on the degradation of methylene blue (MB) is shown in Fig. 7. This organic compound (MB structure, inset Fig. 7a) is a compound used as an organic probe molecule, considered to be a textile product model compound with dye characteristics. Figure 7 shows the concentration of methylene blue over the reaction time in the presence of catalysts (CT-Fe, CT-FeCr and pure magnetite) at different temperatures.

According to the results shown in Fig. 7, it was observed from the kinetic degradation profiles of the CT-Fe and magnetite (Fe_3_O_4_) materials that there was no significant degradation of the MB during the 240 min of reaction at temperatures of 25, 40, and 60 °C. At 80 °C, CT-Fe and Fe_3_O_4_ showed degradations of approximately 5.43% and 14.9%, respectively at the end of 240 min of reaction. Interestingly, the control test (H_2_O_2_ + dye, without catalyst) had an MB decolorization rate of 42.6%, higher than the systems with the presence of the catalysts CT-Fe and Fe_3_O_4_. To explain the results, it should be remembered that the Fenton process is based on the activation of H_2_O_2_ according to the mechanism proposed by Haber-Weiss^39^ to form the radical HO∙^12,40,41^:

1 $${{\rm{Fe}}}^{2+}+{{\rm{H}}}_{2}{{\rm{O}}}_{2}\to {{\rm{Fe}}}^{3+}+{{\rm{HO}}}^{-}+{\rm{HO}}\,\cdot \,({{\rm{k}}}_{1}=63\,{{\rm{mol}}}^{-1}{\rm{L}}\,{{\rm{s}}}^{-1})$$

2 $${{\rm{Fe}}}^{3+}+{{\rm{H}}}_{2}{{\rm{O}}}_{2}\to {{\rm{Fe}}}^{2+}+{{\rm{HO}}}_{2}\,\cdot +{{\rm{H}}}^{+}\,({{\rm{k}}}_{2}=0,002-0.01\,{{\rm{mol}}}^{-1}\,{\rm{L}}\,{{\rm{s}}}^{-1})$$

3 $${\rm{HO}}\,\cdot +{\rm{MB}}\to {\rm{intermediates}}\to {{\rm{CO}}}_{2}+{{\rm{H}}}_{2}{\rm{O}}+{\rm{oxidized}}\,{\rm{byproducts}}$$

In the presence of an organic substrate (MB) in Eq. (3), the hydroxyl radical abstracts an MB hydrogen atom and generates an organic radical (MB∙), which subsequently undergoes a series of chemical transformations to form several oxidation products (intermediates). In the absence of any competitive elimination of HO∙ or MB∙, the use of excess concentration of Fe^2+^ and H_2_O_2_ should, in principle, completely convert the organic molecule to CO_2_, water and inorganic salts. However, the high nonspecific reactivity of the HO∙ radical for organic and inorganic substrates results in several competitive processes that negatively affect the Fenton process as shown in Eqs (4–6):

4 $${{\rm{Fe}}}^{2+}+{\rm{HO}}\,\cdot \to {{\rm{Fe}}}^{3+}+{{\rm{HO}}}^{-}\,({\rm{k}}=3,2\times {10}^{8}\,{{\rm{mol}}}^{-1}\,{\rm{L}}\,{{\rm{s}}}^{-1})$$

5 $${{\rm{H}}}_{2}{{\rm{O}}}_{2}+{\rm{HO}}\,\cdot \to {{\rm{HO}}}_{2}\,\cdot +{{\rm{H}}}_{2}{{\rm{O}}}_{2}\,({\rm{k}}=3,3\times {10}^{7}\,{{\rm{mol}}}^{-1}\,{\rm{L}}\,{{\rm{s}}}^{-1})$$

6 $${\rm{HO}}\,\cdot +{\rm{HO}}\,\cdot \to {{\rm{H}}}_{2}{{\rm{O}}}_{2}\,({\rm{k}}=6,0\times {10}^{9}\,{{\rm{mol}}}^{-1}\,{\rm{L}}\,{{\rm{s}}}^{-1})$$

To improve the oxidation efficiency, several modifications of the Fenton reaction were performed and classified as Fenton-like or Fenton-like processes^12,41^. Thus, the CT-FeCr beads showed higher MB degradation at all evaluated temperatures. The presence of Cr strongly increased the catalytic activity of the oxide. Combined with the catalytic gain from the incorporation of the second metal in the hybrid composite structure, the kinetics of MB degradation was favored by increasing temperature, considering that MB degradation at 80 °C was 97.60% after only 15 min of reaction. However, Liao and coworkers^42^ have already mentioned the antagonistic effect achieved in processes based on Fenton chemistry when performed at higher temperatures. On one hand, higher temperatures are shown as favorable parameters for the Fenton chemistry because they favor both hydroxyl radical formation and enhance the regeneration rate of Fe^3+^/Fe^2+^ shown in Eqs (1 and 2); on the other hand, it is also known that temperature values close to or greater than 80 °C tend to facilitate metallic leaching and occurrence of parallel reactions in the homogeneous phase. Furthermore, in Fenton chemistry temperatures greater than or equal to 80 °C, lead to the decomposition of H_2_O_2_, predominantly to H_2_O and O_2_, limiting the formation of those oxidizing groups typical of the advanced oxidation processes that are available for oxidation. This process is shown in Eq. (5)^43^. Although one of the formed products has also a radical characteristic, the resonance of the unpaired electron between the two oxygen atoms greatly diminishes the oxidizing power of the hydroperoxyl radical (HO_2_∙) before the hydroxyl radical (HO∙).

Data shown here demonstrates that the MB degradation kinetics was favored by increasing of temperature, but only for CT-FeCr. Although the catalyst CT-FeCr presents a better performance at 60 °C, the material is efficient to promote catalysis in all temperatures tested by the end of 240 min (degradation MB 64% at 25 °C, 83% at 40 °C, 94% at 60 °C and 98% at 80 °C) (Fig. 1Sa, Supplementary Information Dataset 1). Alternatively, we may present catalyst performance is to display data as dye degradation capacity per grams of the catalyst (mg g^−1^) (Fig. 1Sb Supplementary Information Dataset 1). As can be observed, in all temperature range (from 25 to 80 °C), the catalyst displays a high degradation capacity of MB. Thus, the reaction temperature was maintained at 60 °C for the next analyses, since controlling at this temperature does not show degradation of the organic molecule.

#### Effect of the amount of H_2_O_2_

The effect of the amount of H_2_O_2_ on the degradation is an important parameter since the degradation generally increases with the increase of the amount of H_2_O_2_ due to the greater generation of HO∙ (Eqs 1 and 2) radicals. However, excess H_2_O_2_ may not be advantageous due to the hydroxyl radical elimination effect in which H_2_O_2_ reacts with hydroxyl radicals resulting in the production of the radical HO_2_∙ Eq. (5) or O_2_ molecules Eq. (7) with lower oxidation potentials than that of the hydroxyl radical^23,40^.

7 $${{\rm{HO}}}_{2}\cdot +{\rm{HO}}\cdot \to {{\rm{H}}}_{2}{\rm{O}}+{{\rm{O}}}_{2}$$

**Figure 8:**
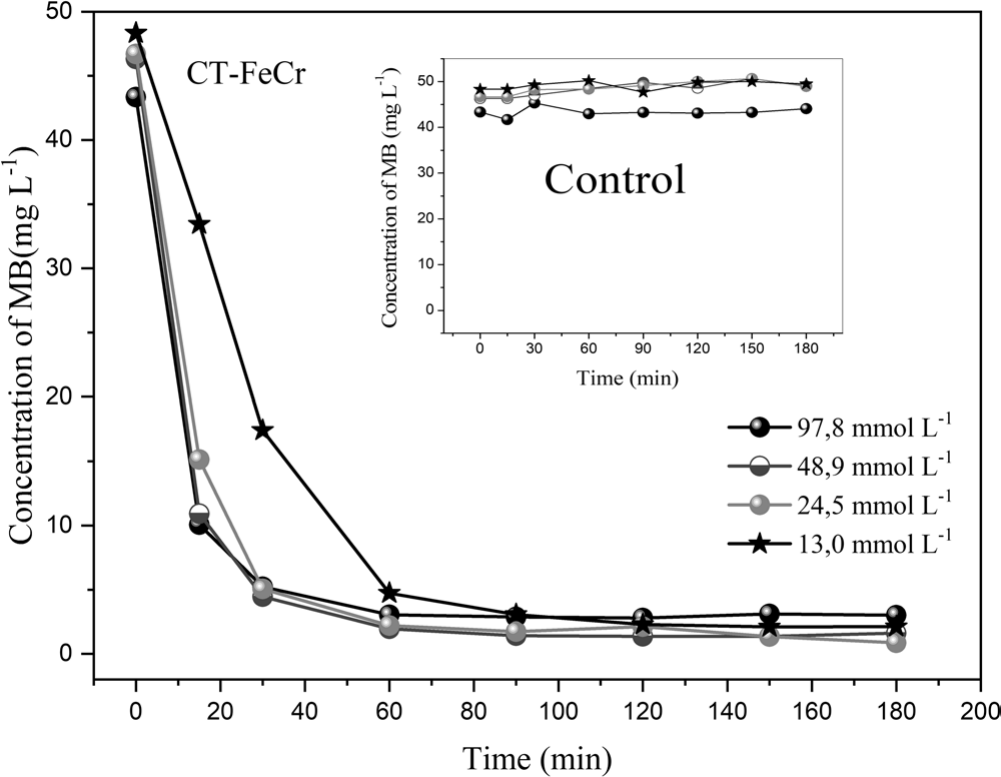
Effect of H_2_O_2_ concentration (13.0 to 97.8 mmol L^−1)^ in methylene blue degradation - MB (1 g L^−1^ CT-FeCr and control (H_2_O_2_ + dye and without catalyst)/MB solution of 50 mg L^−1^ pH 6.0, temperature 60 °C.

Thus, the effect of the amount of H_2_O_2_ on MB degradation was investigated and is presented in Fig. 8.

The results of tests on the variation of oxidant concentration showed that any MB degradation that occurred by CT-Fe and Fe_3_O_4_ materials at lower H_2_O_2_ concentrations was not as efficient in the MB degradation (results not shown). For the CT-FeCr beads, it was observed that the dye degradation was almost the same in 60 min of reaction (93.6% on average of MB degradation) even at lower H_2_O_2_ concentrations.

According to Soon and Hameed^44,45^, the stoichiometry and the chemical *pathway* for the removal of synthetic dyes from aqueous solution are not yet completely clear in the heterogeneous Fenton catalytic process^46^. Therefore, the H_2_O_2_ concentrations used in this study were lower than the concentration of 97.8 mmol L^−1^ because higher H_2_O_2_ concentrations may reduce the degradation efficiency due to the HO∙ elimination^41,47^. This is because the CT-FeCr beads became brittle at the end of the temperature studied, a behavior that was associated with a high oxidant concentration. At H_2_O_2_ concentrations of 24.5 mmol L^−1^ and 13.0 mmol L^−1^, the beads maintained their shape at the end of 180 min of reaction. Thus, the concentration of 13.0 mmol L^−1^ was chosen for the analysis of bead reuse due to the maintenance of the bead shape, besides guaranteeing the degradation efficiency and minimizing the costs associated with the oxidant expense.

#### Reuse of the CT-FeCr beads

**Figure 9:**
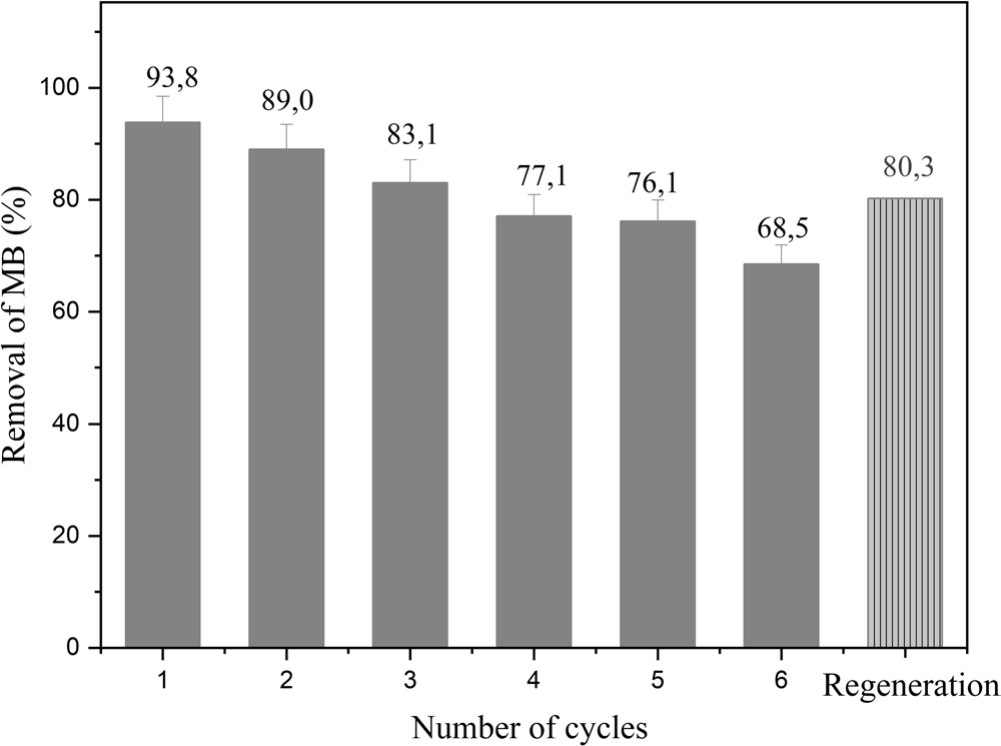
Number of cycles of CT-FeCr beads in the methylene blue - MB degradation (1 g L^−1^ CT-FeCr/solution of MB 50 mg L^−1^, temperature 60 °C, contact time 60 min, 13 mmol L^−1^ H_2_O_2_) and regeneration of the CT-FeCr catalyst after the sixth cycle of reuse.

The reuse of catalysts is of great importance in the evaluation of the technical and operational viability for the implementation of a material with catalytic properties. To investigate the long-term stability of the catalyst, the CT-FeCr beads were used in consecutive 60-min reaction cycles (Fig. 9).

The CT-FeCr beads showed 93.8% of MB degradation efficiency in the first cycle. In the second reaction cycle, the MB removal rate decreased by 5.1% when submitted to a new recharge of the dye. The slightly reduced catalytic activity of the catalyst is expected due to consecutive uses in experiments, which can be attributed to leachate of the active component of the catalyst and/or contamination by intermediates formed during the reaction^23^.

**Figure 10:**
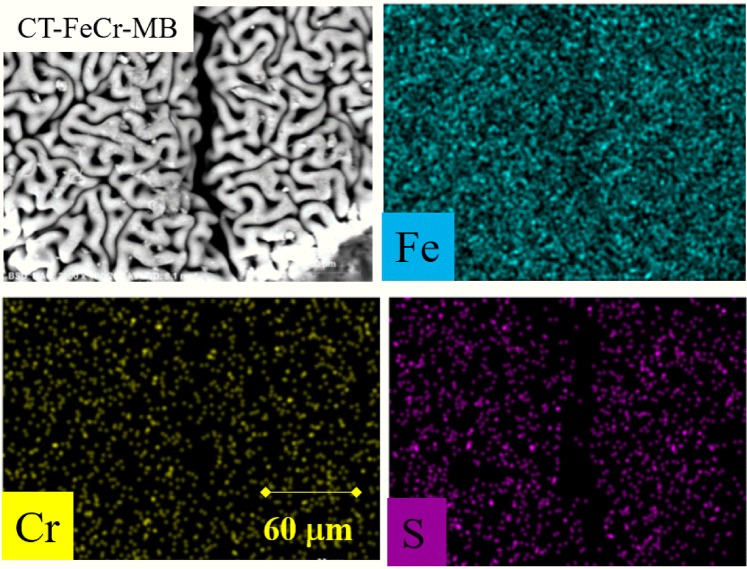
Scanning electron microscopy (SEM) and energy dispersive spectroscopy (EDS CT-FeCr) after methylene blue degradation.

In this study, at the end of each cycle the dosages were measured by colorimetric methods after the reactions in order to investigate the possibility of homogeneous reactions by Fe and Cr leached species. Fe and Cr concentrations were all below the limit of detection of the technique. Therefore, the decrease in catalytic activity after the consecutive cycles can be considered as poisoning for the catalyst active sites, caused by the adsorption of intermediates produced during the catalytic reaction. This becomes more evident with the SEM results coupled to EDS analysis (Fig. 10).

The morphological analysis of the CT-FeCr-MB beads showed that the surface retains the roughened and irregular appearance similar to the material characterized prior to the dye degradation (Fig. 2c). Moreover, the elemental analysis profile obtained by the chemical mapping showed the presence of Fe and Cr elements on the catalyst whole surface without the presence of dark zones, which would indicate the absence of metals. These results are the first evidence of the absence of metal leaching.

In addition to these data, the EDS mapping results after the sixth reaction cycle for the CT-FeCr beads presented a profile typically different from that shown in Fig. 2c. In the discoloration kinetics, these materials showed an MB removal efficiency of 68.5% in the sixth cycle of reuse, indicating that an important part of its initial activity was lost. Together with this loss of activity, the beads were monitored by EDS and significant amounts of sulfur could be identified on their surface. In the MB structure shown in Fig. 7a, the observed S incorporated in the material is possibly related, as predicted by Whang *et al*.^23^, to reaction intermediates that are strongly adsorbed on the catalyst and eventually form the so-called poisons of catalytic sites. The adsorption of these intermediates on the catalyst surface also led to the reduction of approximately 77% of their surface area (Fig. 4a). This poisoning prevents the action of H_2_O_2_, since the active sites decrease with each catalytic cycle.

After the sixth cycle of MB removal, the material was submitted to a proposed regeneration of the activity by an ethyl alcohol catalyst rinsing. After the proposed desorption step of the reaction intermediates, an increase of 11.8% in the activity of the material could be observed, thus obtaining a removal of 80.3% of the MB (Fig. 9). These results demonstrate that the synthesized catalyst shows good reuse performance after catalyst regeneration.

### Proposed mechanism for the CT-FeCr catalyst

The Fe^2+^ present in the CT-FeCr composite structure is extremely important for the heterogeneous Fenton reactions, in which the mechanism of radical formation *via* H_2_O_2_ decomposition follows the Haber-Weiss proposal. These results also suggest that both Cr^6+^ and Cr^3+^ can participate in processes known as Fenton-like, acting as electron donors in order to form HO∙ radicals^18,20,21,48,49^. On the other hand, there is still the possibility of kinetic gain arising from the greater ease in electron transfer during the Fe^2+^ recovery process^19^. Previous studies have demonstrated that the hexavalent chromium is attracted to the surface of chitosan by electrostatic forces. On the surface, chromium is reduced to Cr^3+^ and posteriorly complexed by covalent bonds to the chitosan structure. Thus, it is believed that the reduced form (Cr^3+^) is present in greater quantity on the CT-FeCr catalyst surface after removal of the aqueous Cr^6+^.

**Figure 11:**
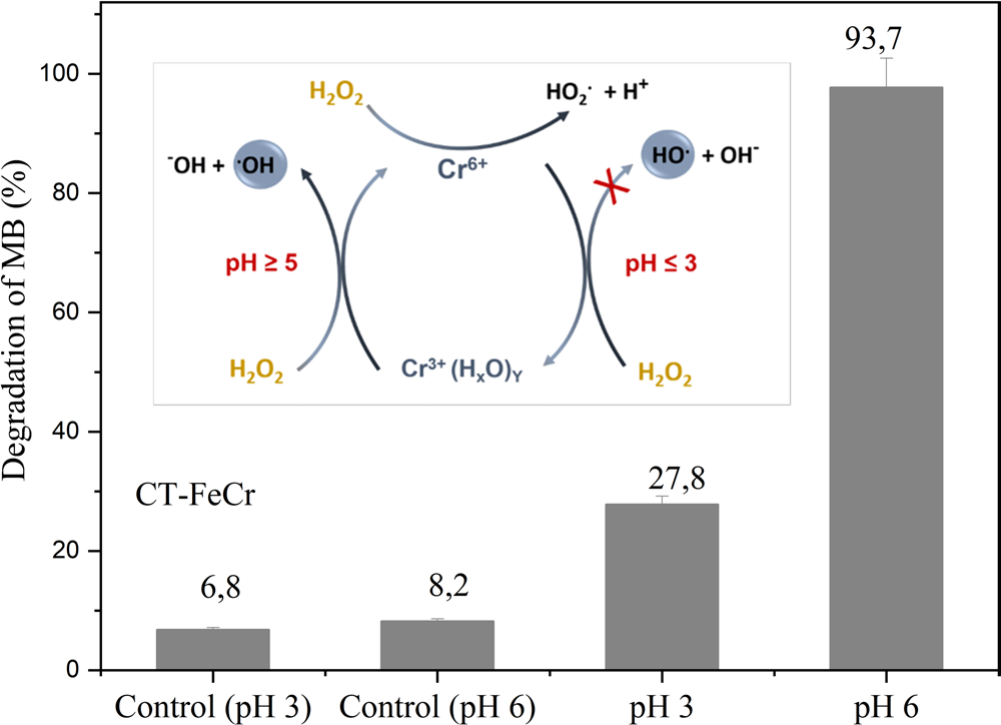
Effect of pH on methylene blue degradation - MB (1 g L^−1^ CT-FeCr and control (H_2_O_2_ + dye and no catalyst)/MB 50 mg L^−1^ solution, temperature 60 °C) and insert AOPs scheme based on redox cycle Cr^3+^/Cr^6+^ in the presence of H_2_O_2_ (adapted from BOKARE *et al*.^21^).

In the case of the Fenton-like process occurring with Cr^3+^, the trivalent form of Cr shows a reaction mechanism with H_2_O_2_ that is strongly pH dependent (Fig. 11, inset). The pH can influence the formation of reactive intermediates as well as the formation of hydroxyl radicals. At pH below 3.0, Cr^3+^ does not react with H_2_O_2_, while presenting maximum reactivity at pH values above 5.0^21,49^. Thus, for a better elucidation of the reaction mechanism, a study of MB degradation by the CT-FeCr beads at pH 3.0 (dye solution adjusted with H_2_SO_4_ 0.5 mol L^−1^) and pH 6.0 (dye solution without pH adjustment) was performed. The result is shown in Fig. 11.

The results show that the MB degradation by the CT-FeCr catalyst at pH 3.0 reduced its activity by 70.3% in relation to pH 6.0, indicating that the generation of HO∙ radicals can occur with the reaction of the Cr^3+^ present on the catalyst surface and H_2_O_2_. The formation of hydroxyl radicals initiated at pH ≥ 5 is in accordance with reports in the literature in which Cr^3+^ activity is totally inhibited at pH 3.0 and increases with increasing pH. The unexpected pH-dependent reactivity is attributed to the formation of highly reactive Cr^3+^ polynuclear surface species (Fig. 11). These complexes are intermediate species formed during the surface variation induced by pH. All these oligomers react with H_2_O_2_ and generate HO∙, the degree of oligomerization being directly proportional to the increase in pH of the solution^21,49^.

The generation of HO∙ radicals occurs through the formation of reactive chromium intermediates, such as Cr^4+^ and Cr^5+^ by the oxidation of a sequential Cr^3+^ electron to a series of reactions with H_2_O_2_ Eqs (8–10):

8 $${{\rm{Cr}}}^{3+}+{{\rm{H}}}_{2}{{\rm{O}}}_{2}\to {{\rm{Cr}}}^{4+}+{{\rm{HO}}}^{-}+{\rm{HO}}\cdot $$

9 $${{\rm{Cr}}}^{4+}+{{\rm{H}}}_{2}{{\rm{O}}}_{2}\to {{\rm{Cr}}}^{5+}+{{\rm{HO}}}^{-}+{\rm{HO}}\cdot $$

10 $${{\rm{Cr}}}^{5+}+{{\rm{H}}}_{2}{{\rm{O}}}_{2}\to {{\rm{Cr}}}^{6+}+{{\rm{HO}}}^{-}+{\rm{HO}}\cdot $$

In comparison to Cr^3+^, the hexavalent chromium shows a non-pH-dependent reaction mechanism. The generation of radicals follows a sequential *pathway* similar to the Cr^3+^/H_2_O_2_ system of Eqs (11–13).

11 $${{\rm{Cr}}}^{6+}+{{\rm{ne}}}^{-}\to {{\rm{Cr}}}^{5+}\,{\rm{or}}\,{{\rm{Cr}}}^{4+}\,{\rm{or}}\,{{\rm{Cr}}}^{3+}\,({\rm{n}}=1-3)$$

12 $${{\rm{Cr}}}^{5+}+{{\rm{H}}}_{2}{{\rm{O}}}_{2}\to {{\rm{Cr}}}^{4+}+{{\rm{HO}}}_{2}\,\cdot +{{\rm{H}}}^{+}$$

13 $${{\rm{Cr}}}^{4+}+{{\rm{H}}}_{2}{{\rm{O}}}_{2}\to {{\rm{Cr}}}^{3+}+{{\rm{HO}}}_{2}\,\cdot +{{\rm{H}}}^{+}$$

Using H_2_O_2_ as both oxidant and reductant, the oxidation from Cr^3+^ to Cr^6+^ and subsequent regeneration can easily occur with the direct reduction of Cr^6+^ [E° (H_2_O_2_/O_2_) = +0.68 V] and [E^0^ (Cr^6+^/Cr^3+^) = +1.35 V], since each redox conversion [Cr^3+^ → Cr^6+^ and Cr^6+^ → Cr^3+^] is accompanied by the formation of HO∙^21^.

Another reaction mechanism, whose results show that it possibly occurs in parallel, but not to the same extent, is proposed based on the transfer of electrons between the species Cr^3+^, Fe^3+^ and Fe^2+^. In this parallel mechanism, the redox potential values of the metals involved in the material come into play. In it, the Cr^3+^ species activates H_2_O_2_, forming HOO∙, O_2_∙^−^ and Cr^2+^ species, Eqs (14) and (15). Thus, reduced Cr^2+^ ions may participate in the direct formation of hydroxyl radicals in the heterogeneous Fenton reaction, having been shown to be a thermodynamically and kinetically favorable reaction, Eq. (16)^50^.

14 $${{\rm{Cr}}}^{3+}+{{\rm{H}}}_{2}{{\rm{O}}}_{2}\to {{\rm{Cr}}}^{2+}+{\rm{HOO}}\,\cdot +{{\rm{H}}}^{+}$$

15 $${{\rm{Cr}}}^{3+}+{\rm{HOO}}\,\cdot \to {{\rm{Cr}}}^{2+}+{{\rm{O}}}_{2}\,{\cdot }^{-}+{{\rm{H}}}^{+}$$

16 $${{\rm{Cr}}}^{2+}+{{\rm{H}}}_{2}{{\rm{O}}}_{2}\to {{\rm{Cr}}}^{3++}{{\rm{HO}}}^{-}+{\rm{HO}}\,\cdot \,\Delta E=2,184\,{\rm{V}}$$

The presence of chromium in the CT-FeCr beads favors the formation of hydroxyl radicals, which explains the greater potential in relation to the other materials. Furthermore, the formation of Cr^2+^ species can reduce Fe^3+^ to produce active Fe^2+^ species for the iron-activated Fenton-like reaction, Eq. (17).

17 $${{\rm{Fe}}}^{3+}+{{\rm{Cr}}}^{2+}\to {{\rm{Fe}}}^{2+}+{{\rm{Cr}}}^{3+}\,\,\Delta E=1,179\,{\rm{V}}$$

Again, the reaction is thermodynamically and kinetically favorable since the potential of Fe^3+^/Fe^2+^ (0.771 V) is greater than the reduction potential from Cr^3+^ to Cr^2+^ (−0.408 V), therefore the formed Fe^2+^ can generate radicals for MB degradation. The presence of Cr in the CT-Fe beads would be helping in the regeneration of active Fe^2+^ species, which is the most active cation in the Fenton-like reaction^18,20^ and often the process-limiting step, which may prevent the completion of the catalytic cycle.

### Toxicity- hemolysis

**Figure 12:**
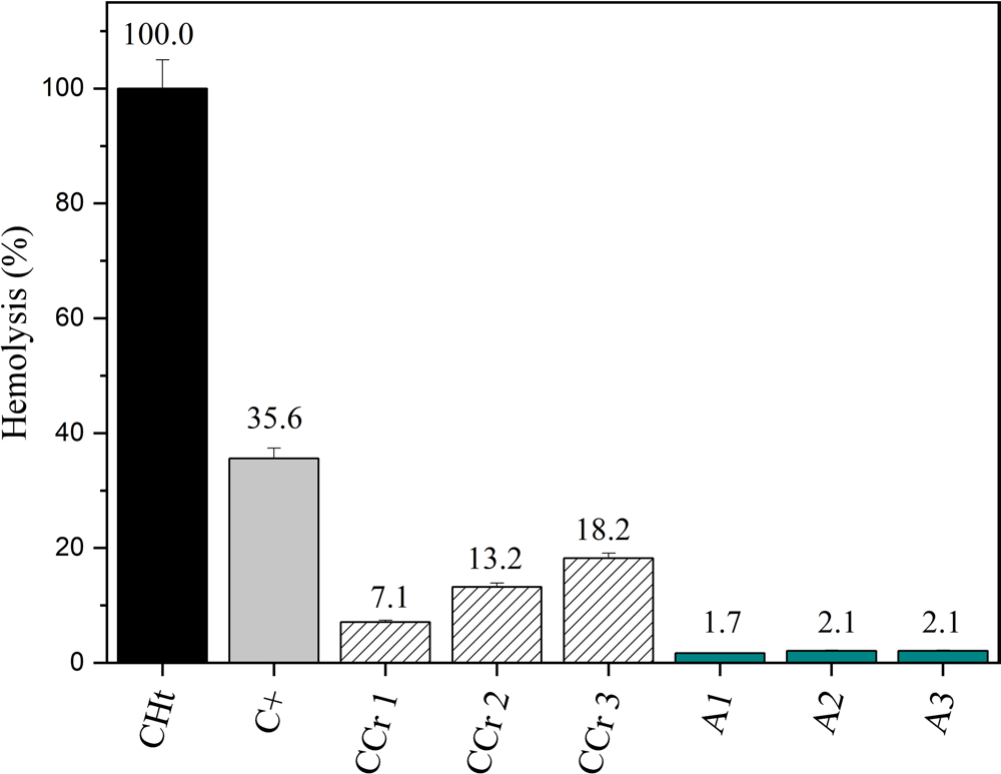
Hemolysis assay performed at a 1% hematocrit. The percentage values provided above the bars in each sample refer to the hemolysis observed. CHt: total hemolysis control; C+: *Bothrops jararacussu*; CCr: Chromium solution control (5 mg L^−1^) A1: supernatant after the degradation of methylene blue.

Hemolysis is an important parameter to assess the safety of substances and materials that were designed to be in direct contact with animal blood cells and tissues or to predict their behavior if they were exposed to these organisms. In this study, we evaluated the effect upon human erythrocytes of the supernatant after the degradation of methylene blue by CT-FeCr beads under experimental conditions. The supernatant was added to a 1% hematocrit in different doses and their hemolytic potential was assessed for 4 h (Fig. 12). According to the ASTM practice guide F 756, samples presenting up to 5% of hemolysis, are considered nonhemolytic. The effects of mechanic hemolysis caused by the manipulation (0.38%; p > 0.05) during the experiment were discounted from the results of all samples.

Past works have discussed the toxicity of chromium, reporting renal impairment, liver dysfunction, neurotoxicity, an increase in reactive oxygen species, hemolysis and genotoxicity^51^. To assess the hemolytic potential of the catalyst CT-FeCr, a chromium standard solution was added in different proportions to isolated erythrocytes (CCr 1, CCr 2 and CCr 3). As a total hemolysis control, erythrocytes were put in ultrapure water and a positive control was prepared from *Bothrops jararacussu* venom (a known hemolytic snake venom). In comparison to the positive control all samples were considered significantly different and presented values below 3%. In the other hand, the chromium standard solution in the evaluated proportions presented hemolysis values of 7%, 13% and 18% (CCr 1, CCr 2 and CCr 3, respectively). Based on the chromium controls, we believe that if there is any release of chromium from the catalyst CT-FeCr, this occurs at a nonhemolytic concentration.

## Conclusions

The proposal for a continuous adsorption-catalysis cycle is successful, since the chromium removed by adsorption in a first step becomes a promoter in the catalytic cycle in the next step. The heavy element Cr, a highly hazardous residue, has immobilized functionality in the catalyst structure and exhibits considerable potential in the MB degradation.

The MB degradation kinetics is favored by an increase in temperature and by the presence of Cr in the beads (CT-FeCr). The reduced form (Cr^3+^) present on the bead surface is fundamental for the increased catalytic activity of iron oxide. This increase in degradation is related to Cr^3+^/Cr^6+^ redox reactions in Fenton-like processes involving Cr^4+^ and Cr^5+^ reactive intermediates dependent of the pH of solution. The double role of H_2_O_2_ as an oxidizing agent of Cr^3+^ and Cr^6+^ reductant can maintain the stability of the Cr^3+^-Cr^6+^-Cr^3+^ redox cycle. The radical formation can also be related to the transfer of electrons by the coupling of Fe^2+^/Fe^3+^ redox pairs with Cr^3+^/Cr^2+^, to which they can regenerate the active Fe^2+^ species more efficiently. This material is also stable, without the release of the active phases of Fe or Cr, with the exclusive catalysis in heterogeneous phase.

In addition, the CT-FeCr beads showed catalytic stability after several consecutive reaction cycles. The beads were demonstrated to be an excellent alternative with technical and economic viability for the treatment of effluents. Furthermore, the product of the catalytic treatment and the very presence of the catalyst itself in the environment do not pose toxic effects. Above all, this technology is of great environmental appeal to the sequential remediation of real inorganic-organic characteristic problems, which makes the proposal green and highly sustainable.

## Supplementary information

Supplementary Information Dataset 1

Video
