# Supplementary material for: Use of an Environmental Pollutant From Hexavalent Chromium Removal as a Green Catalyst in The Fenton Process
Source: Sci Rep. 2019 Sep 6;9:12819. doi: 10.1038/s41598-019-49196-9 (PMC6731299; doi:10.1038/s41598-019-49196-9)
Supplement: Supplementary file 1 — Supplementary Information Dataset 1 [file 41598_2019_49196_MOESM1_ESM.docx]

**SUPPLEMENTARY INFORMATION**

**USE OF AN ENVIRONMENTAL POLLUTANT FROM HEXAVALENT CHROMIUM REMOVAL AS A GREEN CATALYST IN THE FENTON PROCESS**

Pricila Maria Batista Chagas^a^, Aline Aparecida Caetano^a^, Aline Auxiliadora Tirele^b^, Pedro Henrique Souza Cesar^c^, Angelita Duarte Corrêa^c^, Iara do Rosário Guimarães^a*^

^a^ Laboratório de Catálise Ambiental e Novos Materiais, Departamento de Química, Universidade Federal de Lavras, CEP 37200-000, Lavras, MG, Brazil.

^b^ Laboratório de Química, Departamento de Química, Universidade Federal de Itajubá, Centro Universitário de Itajubá, CEP 37500-903, Itajubá, MG, Brazil.

^c^Laboratório de Bioquímica, Departamento de Química, Universidade Federal de Lavras, ZIPCODE 37200-000, Lavras, MG, Brazil

*Corresponding author

Iara do Rosário Guimarães

Laboratório de Catálise e Novos Materiais, Department of Chemistry, Federal University of Lavras

CEP 37200-000, Lavras, MG, Brazil.

Phone: +(55) 35 38291626

E-mail: iaraguimaraes@dqi.ufla.briaraguimaraes@dqi.ufla.br

**Temperature effect**

Figura 1S Effect of temperature on the oxidative degradation of methylene blue – MB, a) Degradation MB (%) b) Degradation MB (mg g^-1^).
